# Spatial Transcriptomic Analysis Using R-Based Computational Machine Learning Reveals the Genetic Profile of Yang or Yin Deficiency Syndrome in Chinese Medicine Theory

**DOI:** 10.1155/2022/5503181

**Published:** 2022-03-16

**Authors:** Cheng Zhang, Chi wing Tam, Guoyi Tang, Yuanyuan Chen, Ning Wang, Yibin Feng

**Affiliations:** School of Chinese Medicine, Li Ka Shing Faculty of Medicine, The University of Hong Kong, Hong Kong, China

## Abstract

**Objectives:**

Yang and Yin are two main concepts responsible for harmonious balance reflecting health conditions based on Chinese medicine theory. Of note, deficiency of either Yang or Yin is associated with disease susceptibility. In this study, we aim to clarify the molecular feature of Yang and Yin deficiency by reanalyzing a transcriptomic data set retrieved from the GEO database using R-based machine learning analyses, which lays a foundation for medical diagnosis, prevention, and treatment of unbalanced Yang or Yin.

**Methods:**

Besides conventional methods for target mining, we took the advantage of spatial transcriptomic analysis using R-based machine learning approaches to elucidate molecular profiles of Yin and Yang deficiency by reanalyzing an RNA-Seq data set (GSE87474) in the GEO focusing on peripheral blood mononuclear cells (PBMCs). The add-on functions in R including GEOquery, DESeq2, WGCNA (target identification with a scale-free topological assumption), Scatterplot3d, Tidyverse, and UpsetR were used. For information in the selected GEO data set, PBMCs representing 20,740 expressed genes were collected from subjects with Yang or Yin deficiency (*n* = 12 each), based on Chinese medicine-related diagnostic criteria.

**Results:**

The symptomatic gene targets for Yang deficiency (KAT2B, NFKB2, CREBBP, GTF2H3) or Yin deficiency (JUNB, JUND, NGLY1, TNF, RAF1, PPP1R15A) were potentially discovered. CREBBP was identified as a shared key contributive gene regulating either the Yang or Yin deficiency group. The intrinsic molecular characteristics of these specific genes could link with clinical observations of Yang/Yin deficiency, in which Yang deficiency is associated with immune dysfunction tendency and energy deregulation, while Yin deficiency mainly contains oxidative stress, dysfunction of the immune system, and abnormal lipid/protein metabolism.

**Conclusion:**

Our study provides representative gene targets and modules for supporting clinical traits of Yang or Yin deficiency in Chinese medicine theory, which is beneficial for promoting the modernization of Chinese medicine theory. Besides, R-based machine learning approaches adopted in this study might be further applied for investigating the underlying genetic polymorphisms related to Chinese medicine theory.

## 1. Introduction

Yin and Yang in Chinese medicine theory account for the two opposing but complementary forces in matters of nature in an interdependent manner [1]. In accordance with Chinese medicine theory, Yang and Yin are considered fundamental for regulating biological processes. The dynamic equilibrium between Yin and Yang is essential for maintaining a healthy body constitution [2]. Accumulating evidence has suggested that Yang-Yin disharmony is a potential inducer of diseases [3]. Cold intolerance develops in patients with Yang deficiency. It is manifested by the coldness in hands or feet, stomach, and waist. In contrast, heat intolerance featured by hyperthermia and xerophthalmia is an indicator of Yin deficiency. Cold hands and waist (cold intolerance) are syndromes of Yang deficiency, while hot body and xerophthalmia (heat intolerance) are indicators of Yin deficiency [4]. Although modern medical studies have identified numerous genetic profiles for specific diseases, underlying genetic polymorphisms related to body susceptibility (Yin or Yang deficiency) in a healthy person remain obscure. The clinical value to investigate the specific genetic profile is as follows: (1) the understanding of genetic profile has beneficial effects on the molecular classification of body constitution of Yin or Yang deficiency; (2) potential genetic regulation of body constitution has important implications for detecting disease-susceptible individuals, which may accelerate the development of precision medicine and health promotion; and (3) a series of Chinese herbs have evidenced to improve Yin or Yang deficiency, such as Radix Ophiopogonis (Mai dong in Chinese) for treating Yin deficiency and Cortex Cinnamomi (Rou gui in Chinese) for Yang deficiency. Our findings may provide a TCM theory-related molecular basis for discovering the therapeutic mechanism of herbal medicines, which may be conducive to the modernization of both TCM theory and Chinese herbal medicine therapy. Therefore, it is worthy of clarifying the functional genetic profile for Yin or Yang deficiency, including key regulatory genes and gene modules.

Circulating peripheral blood mononuclear cells (PBMCs) are composed of lymphocytes (T, B, and NK cells), monocytes, and a small proportion of dendritic cells, showing their potential utility representing the biological activities of our body [5, 6]. It is documented that specific differentially expressed genes in the PBMCs of subjects with or without Yin/Yang deficiency demonstrate the relationship between clinical relevance and responsible functional genes. For instance, a significant increase of thyroid hormone receptor *β* shows molecular interpretations for cold intolerance in Yang deficiency, while the decrease of lipid syntheses, such as ACSL1 and ABCA1, is the indicator of heat intolerance in Yin deficiency [7, 8]. These reports suggested that the underlying molecular profile of Yang and Yin deficiency may be deciphered by genomic analysis on the serum PBMCs. Therefore, we retrieved an RNA-Seq data set (GEO87474) from GEO data set, in which 20,740 genes expressed in PBMCs from subjects with Yang or Yin deficiency (*n* = 12 each) were collected. All the classification of individuals with Yang or Yin deficiency in this data set was based on Chinese medicine-related diagnostic criteria of body constitution [8, 9]. Furthermore, machine learning approaches will be adopted for transcriptomic analysis of this data set. R language-based weighted gene coexpression analysis (WGCNA) is the main analytic approach for target identification. The merit of WGCNA is that it identifies the targets in a scale-free network without a specific cut-off value, in which real-world biological relevance generally represents scale-free behavior [10, 11].

**Figure 1:**
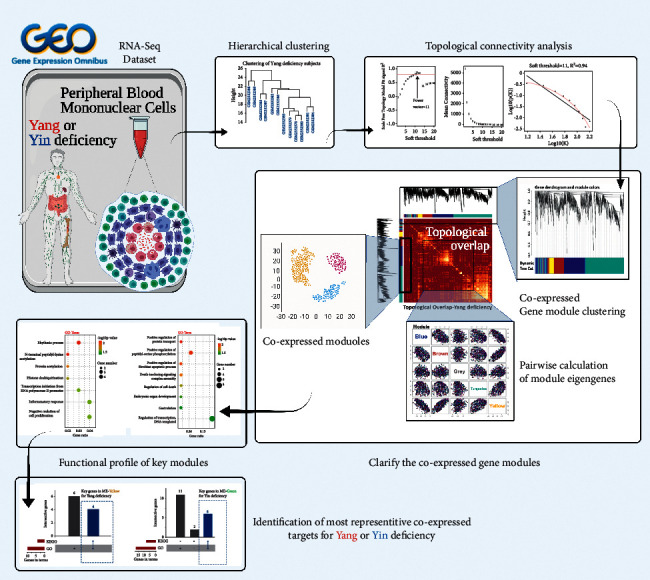
Schematic for the identification of key coexpressed targets in body constitution with either Yang or Yin deficiency.

As an analytical decision-making tool in computer science, R language programming-based arithmetic analysis is ideal for clarifying the genetic polymorphisms of Yang and Yin deficiency. R contains a wide variety of statistical techniques for life science, such as WGCNA and high-dimensional multivariate statistical analysis [12, 13]. Moreover, R is user-friendly and equipped with wide-ranging functionalities with add-on packages available from CRAN (more than 12,000 packages). This makes R more versatile than other statistical approaches in data manipulation. In accordance with the workflow (Figure 1), in this study, taking the advantage of R-based functions, we provided a scientific and computational identification of representative targets for Yang and Yin deficiency, which may promote the modernization of Chinese medicine theory.

## 2. Methods

### 2.1. Data Set Acquisition and Normalization

A clinical data set consisting of an RNA-Seq data set (GSE87474) was retrieved from the GEO database, collecting 20,740 genes expressed in PBMCs from subjects with Yang or Yin deficiency (*n* = 12 each). The classification of individuals with Yang or Yin deficiency was determined by Professor Wang Qi's Body Constitution Classification Questionnaire, which is based on Chinese medicine theory [8]. The main clinical symptoms for Yang or Yin deficiency are listed in Table 1.

The RNA-Seq data set from distinctive groups (Yin or Yang deficiency) was analyzed separately. The inclusion criteria of recruited individuals in GSE87474 are as follows: (1) healthy individuals with Yang or Yin deficiency constitution were determined by a standardized questionnaire for the body constitution classification created by Wang Qi [8] and (2) 18–28 years old. Meanwhile, the exclusion criteria are as follows: (1) individuals are not related to Yang or Yin deficiency resulted from the body questionnaire; (2) beyond or below 18–28 years old; and (3) no medical records of diagnosed diseases.

For retrieving the data set on the platform of Affymetrix HG-U133A Plus 2.0 array, the R package “GEOquery” was used to download the GSE87474. The Affymetrix annotation package for human termed “hugene10sttranscriptcluster.db” was adopted for matching probes to corresponding gene symbols [14]. Afterwards, the raw data of transcriptome-wide gene expressions in subjects with Yang or Yin deficiency were obtained for further analysis. For the normalization of raw data, the DESeq2 algorithm in R was used for a variance-stabilizing transformation [15]. Simultaneously, the outliers were removed upon Cook's distance by DESeq2 calculation as well. Herein, the normalized data set summarized in Supplementary Data 1 was used for gene coexpression analysis.

### 2.2. Data Filtering

For WGCNA-based data filtering, the R function “apply ()” in WGCNA was adopted for ranking the variance of gene expressions in either Yin or Yang deficiency. Those expression variances larger than 90^th^ percentile of the whole genome were selected. Finally, 2016 genes in either Yang or Yin deficiency were left as the most informative ones for both body constitutions for WGCNA, including scale-free topology assumption and screening key coexpressed genes/modules.

### 2.3. Sample Clustering and WGCNA (Key Genes and Modules)

For data clustering and module construction, the R function “hclust ()” was used to establish a homogeneous sample microarray from either Yang or Yin data sets. Similar data will be grouped in a consensus branch site. The similarity of the consensus matrix was identified by calculating the coefficients of Pearson's correlation s_*ij*_ = cor(*i*, *j*) of the whole gene set. Afterwards, a weighted adjacency matrix was established for calculating coexpression similarity by an equation: *a*_*ij*_ = |(1 + cor(*i*, *j*))/2|^*β*^, in which *β* is a soft thresholding power (a threshold parameter for weighted analysis), while a_ij_ represents the resulting adjacency evaluating the strength of weighted connectivity [16]. Finally, a dendrogram of classifying consensus gene modules of gene expression and a topological overlap matrix (TOM) were plotted based on the value of module eigengenes (MEs). The result of MEs is calculated upon the first principal component of the gene expression matrix of gene modules by principal component analysis (PCA) [17]. The filtered key genes with its modules upon the value of ME are shown in Supplementary Data 2 (Yang deficiency group) and Supplementary Data 3 (Yin deficiency group). Besides, both intermodular Pearson's *r* and *p* values provided by the Kruskal–Wallis test and one-way ANOVA with Tukey's multiple comparison test were calculated for measuring eigengene correlation. Moreover, the relationships among coexpressed intermodules can be shown by multidimensional views. The function scatter analysis by R was used for evaluating the positions of hub gene modules. The identification of key gene module was based on the sum value of the *p* value among each gene module with its correlated modules. The gene module with the minimum cumulative *p* value was selected as the most representative module for its corresponding group (Yang or Yin deficiency group). The key genes for the most representative module are identified and ranked by its value of soft connectivity (gene connectivity in a weighted adjacency matrix) in a module, which are functionally significant and usually in the center location of a module.

### 2.4. Functional Annotation Analysis and Key Functional Gene Identification

In order to understand the module-associated biological functions, both gene ontology (GO) and Kyoto Encyclopedia of Genes and Genomes (KEGG) enrichment analysis were adopted to analyze the biological relevance of modules [18]. The results further offer valuable insights into Yang and Yin deficiency in subjects. The annotation analysis was performed by an online database DAVID (https://david.ncifcrf.gov/tools.jsp). A *p* value less than 0.05 was regarded as significant. For the functional key genes, the R function UpsetR was used to identify the cross-linked core functional genes for coexpressed gene modules.

## 3. Results

### 3.1. Constructing Weighted Coexpressed Correlation of Gene Modules

**Figure 2:**
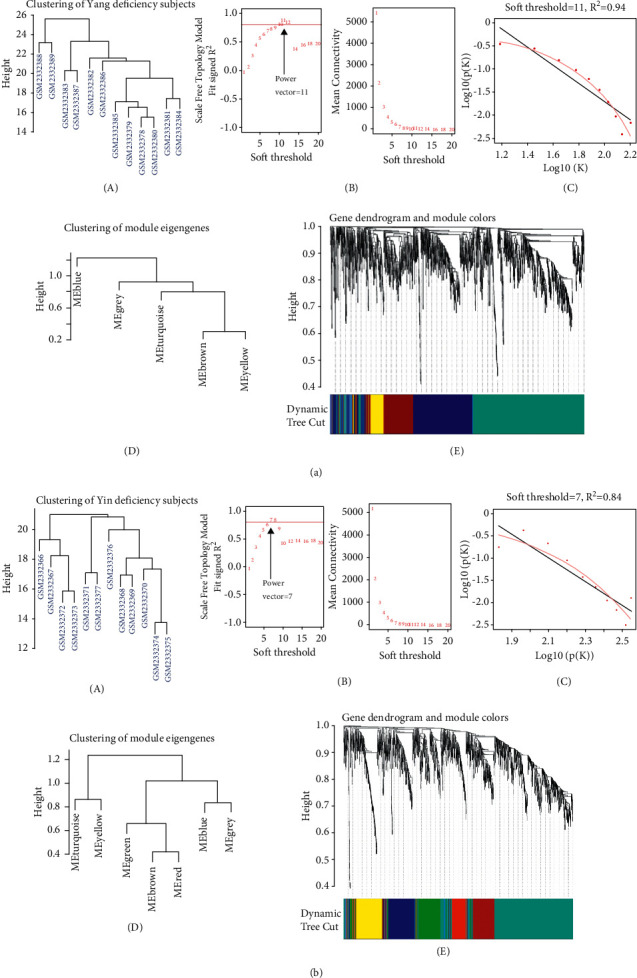
Hierarchical clustering and topological connectivity analysis. (a and b) Clustering of genes from PBMCs in either the Yang or Yin deficiency group by WGCNA. (A) is the hierarchical clustering plot. (B) shows the result of power vector selection. (C) indicates the log-log plot of connectivity of signed adjacency matrices. (D and E) are the gene clustering dendrogram. Sections with different colors under the dendrogram stand for the identified gene modules.

To identify highly correlated gene modules, WGCNA was used to establish the coexpression modules of biological relevance. Data acquisition and normalization for GEO data GSE87474 were conducted in terms of the Methods part. After obtaining the filtered 90^th^ percentile of the whole genome in either the Yang or Yin deficiency group, around 2016 genes in each group (*n* = 12 in each group) were adopted for hierarchical clustering (Figures 2(a) and 2(b)). The scale-free clustering dendrogram was constructed with *β* = 11 (for Yang deficiency) and *β* = 7 (for Yang deficiency). The values of scale-free topology fit signed *R*^2^ in both networks are all larger than 0.8 (*R*^2^ = 0.94 for Yang deficiency and *R*^2^ = 0.84 for Yin deficiency), suggesting the successful construction of scale-free correlations. Thus, 5 and 7 gene modules were identified in Yang or Yin deficiency groups, respectively, which are as follows: 5 modules (ME-blue, ME-grey, ME-turquoise, ME-brown, and ME-yellow) in Yang deficiency and 7 modules (ME-turquoise, ME-yellow, ME-green, ME-brown, ME-red, ME-blue, and ME-grey) in Yin deficiency (Figures 2(a) and 2(b)). Therefore, the WGCNA-related representative gene modules were established and used for further target identification.

### 3.2. Identification of Responsible Coexpression Gene Modules

**Figure 3:**
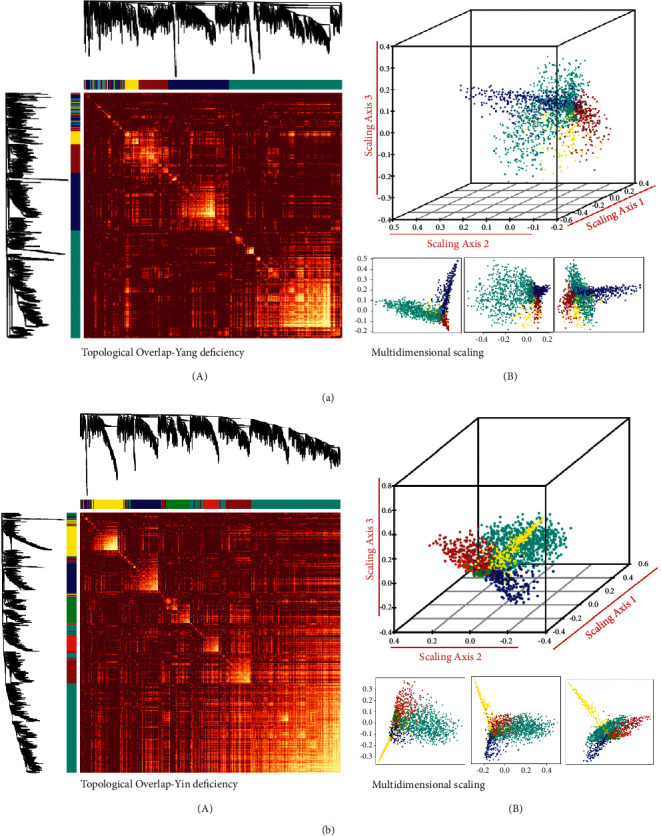
Topological visualization of coexpression genes in modules. (a and b) Topological overlap plot of filtered genes, in which the included data are variances larger than 90^th^ percentile of the whole genome (A) and (B) illustrates the geometric interpretation of gene expression in a 3D scatter plot.

**Figure 4:**
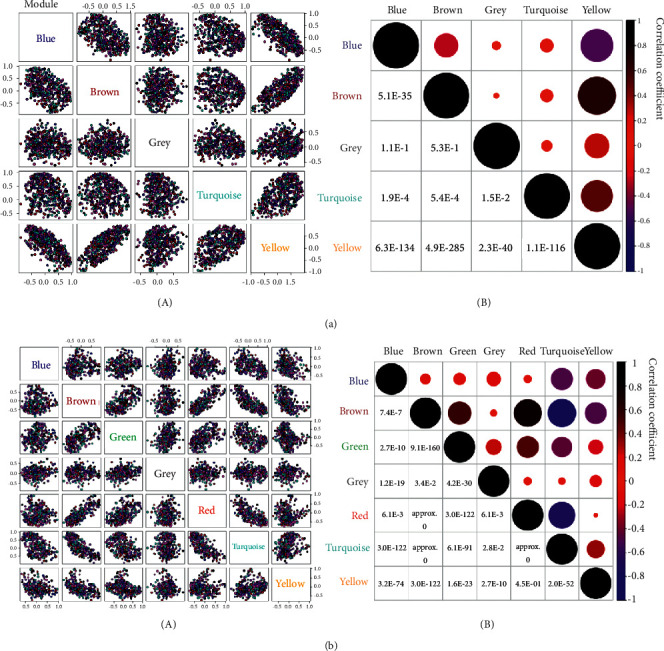
Pairwise analysis of gene modules based on the value of module eigengenes. (a and b) Pairwise scatterplot of identified gene modules by WGCNA (A). In B, the correlation plot indicates the intermodular Pearson's *r* (upper panel with bubbles) and *p* value (lower panel with specific values) provided by the Kruskal–Wallis test and one-way ANOVA with Tukey's multiple comparison test.

Based on the value of module eigengenes calculated by PCA, a topological overlaps-based hierarchical cluster was conducted to classify the coexpressed genes with high similarity, as shown in Figures 2(a) and 2(b). Furthermore, taking the advantage of geometric interpretation for data manipulation, we used a scatter plot to illustrate gene expression upon the value of module eigengenes in multiple dimensions. The results suggested that the intramodular key genes were distributed into separated locations (Figures 3(a) and 3(b)). Prompt by these findings, we continuously calculated the relationship between gene modules, including Pearson's *r* and *p* values. The pairwise plot of module correlation is shown in Figures 4(a) and 4(b). According to the minimum sum of one module with other ones, both ME-yellow in Yang deficiency (cumulative *p* value = 2.3*E* − 40; all *p* value of ME-yellow with other modules <0.001) and ME-green in Yin deficiency (cumulative *p* value = 2.7*E* − 10; all *p* value of ME-green with other modules <0.001) were selected as the most representative module for each body symptom.

### 3.3. Determination of Top Hub Genes in Representative Gene Modules

**Figure 5:**
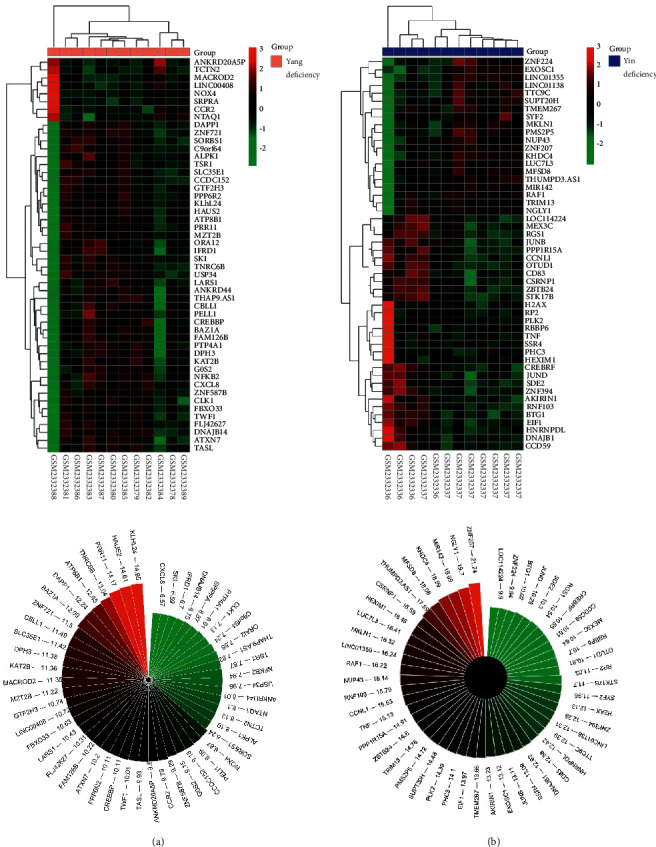
Heatmap for top 50 targets of representative gene modules in distinctive body constitution. (a and b) In terms of the intramodular soft connectivity of genes, the top 50 gene targets in ME-yellow of the Yang deficiency group and ME-green of the Yin deficiency group are shown in the heatmap and Nightingale's rose map.

After confirming the dominant gene module in either Yang (ME-yellow) or Yin deficiency (ME-green) body constitution, we further identified the hub genes in each module. In terms of the intramodular soft connectivity of genes, the top 50 gene targets were shown in the heatmap and Nightingale's rose map using R functions “dplyr” and “Pheatmap” (Figures 5(a) and 5(b)). The soft connectivity of each gene in its corresponding module is indicated beside the gene term, suggesting the weighted value of genes. In sum, the key targets in the representative module of each body constitution were identified. The functional annotation of ME-yellow in Yang deficiency and ME-green in Yin deficiency would be determined by these top 50 genes in each module.

### 3.4. Functional Annotation of Key Modules and Responsible Functional Genes

**Figure 6:**
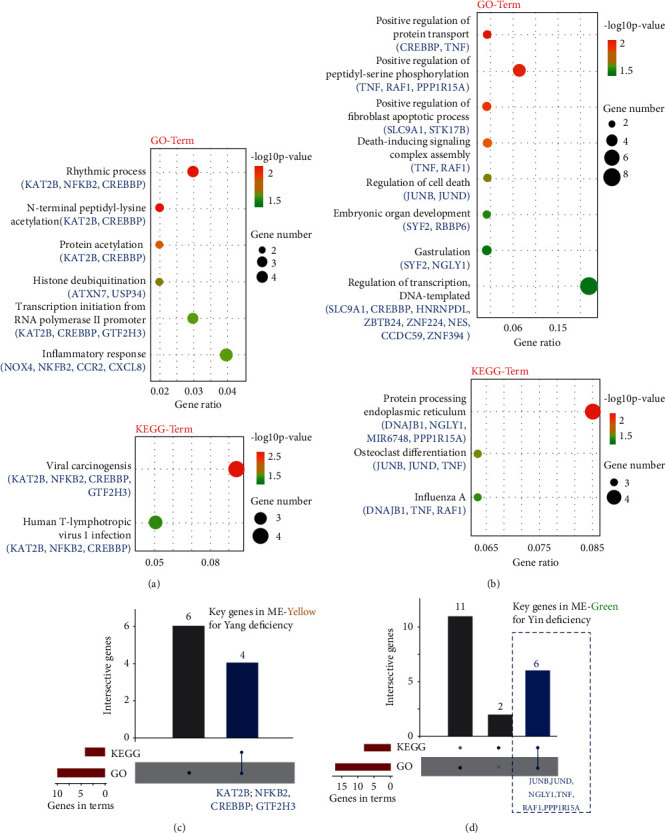
Functional enrichment analysis of gene modules and key targets. (a and b) GO and KEGG functional enrichment analysis for top 50 genes in either the Yang or Yin deficiency group. (c and d) The UpsetR-related column plot illustrates the interactive key functional coexpressed targets in gene modules by GO and KEGG analysis in the same group (Yang or Yin deficiency group).

The functional analysis of each module was performed in the database of gene ontology (GO) and Kyoto Encyclopedia of Genes and Genomes (KEGG). As shown in Figures 6(a) and 6(b), the most enriched functional terms in either the Yang or Yin deficiency group are as follows: For the ME-yellow module in the Yang deficiency group, GO terms “rhythmic process (*p* value = 0.0059)” and “N-terminal peptidyl-lysine (*p* value = 0.0064)” were the most enriched ones, while “viral carcinogenesis (*p* value = 0.0019)” and “human T-lymphotropic virus 1 infection” were the dominant terms in KEGG analysis (*p* value = 0.0019)”. For ME-green in the Yin deficiency group, GO terms “positive regulation of protein transport (*p* value = 0.0090)” and “positive regulation of peptidyl-serine phosphorylation (*p* value = 0.0010)” were the most responsible functions, while “protein processing in endoplasmic reticulum (*p* value = 0.0004)” and “osteoclast differentiation (*p* value = 0.0281)” were the key terms in KEGG analysis. Additionally, we further analyzed the genes for the construction of these functional terms using the R function UpsetR, aiming to identify whether the underlying genes for these GO or KEGG terms were homogeneous. As shown in Figures 6(a) and 6(b), the key genes in ME-yellow of the Yang deficiency group were KAT2B, NFKB2, CREBBP, and GTF2H3, while JUNB, JUND, NGLY1, TNF, RAF1, and PPP1R15A were the key genes in ME-green of the Yin deficiency group. These identified hub genes might be beneficial for medical diagnose, prevention, and treatment of excessive of either Yang or Yin in body constitution.

## 4. Discussion

Distinctions between subjects with or without normal physiological body function are of substantial interest in life science. Although the pathological changes of gene expression in response to various diseases have been extensively investigated, it is still obscure whether normal individuals with specific body constitution are susceptible to diseases. Previous reports have shown that the difference in gene expression of PBMCs from healthy individuals is linked with clinical significance. However, inadequate research studies have investigated the correlation between physiological features and disease susceptibility. Of note, Chinese medicine theory has a conceptual framework to classify healthy people with disease risks with Yin-Yang theory, which informs as a diagnostic role in Chinese medicine therapy [19]. Yin-yang harmony points to the healthy status, whereas the imbalance in either side could lead to the generation of diseases [20]. Following this opinion and regarding the current COVID-19 pandemic, we suggested that maintaining the Yin-Yang balance may also be beneficial for reducing the susceptible individuals to SARS-CoV-2 infection. Therefore, discovering the molecular profile of body constitution is beneficial for health promotion.

Unlike most conventional approaches, we use R-based WGCNA for target and gene module identification followed by understanding the genomic profile of Chinese medicine theory (Yang or Yin deficiency). The advantage of computational pharmacology on reanalyzing the genomic data of distinctive body constitutions has four aspects: (1) it allows rapid and comprehensive observation of genomic profile; (2) the lack of preliminary hypothesis in an unsupervised analysis improves the reliability of the outcomes; (3) the results may exceed the typical understanding of our knowledge and provide discoveries; and (4) conventional analyses mainly use a cut-off value to distinguish the strength of target or module connections, especially the differential expression between groups by an unweighted analysis, which could lead to information loss. For instance, if the cut-off value of a biological differentiation is 0.8, it is not fully considered if the biological significance of 0.79 is rejected. However, WGCNA could avoid this drawback by calculating (weighting) the correlation coefficient with a power value, which may meet the scale-free relationship of a real-world network [21]. Therefore, our study may clarify the intrinsic molecular characteristics of both Yang and Yin deficiency by machine learning approaches.

Although these enriched GO and KEGG terms could provide functional insights into the gene modules, the terms in either the Yang or Yin deficiency group were not well consistent in the same group, which may be resulted from the limited sample size and genetic diversity of hub genes. Thus, instead of focusing on the explanation of the functional annotations, we used the R function UpsetR to investigate whether the background genes for these GO or KEGG terms were homogeneous. Surprisingly, the key genes for supporting GO or KEGG terms were highly consistent, suggesting the critical role of these targets for representing the main feature of either Yang or Yin deficiency of body constitution. For the key modules identified in terms of the criteria described in the Methods part, ME-yellow and ME-green were the representative modules for Yang and Yin deficiency groups, respectively. In particular, KAT2B, NFKB2, CREBBP, and GTF2H3 were the key contributive targets for the Yang deficiency group. Histone acetyltransferase KAT2B (lysine acetyltransferase 2B) is a protein-coding gene, which plays a critical role in the transcriptional regulation of adipocyte differentiation [22]. Noncanonical NF-*κ*B pathway signaling is integral in immunoregulation. Mutation of NFKB2 (nuclear factor kappa B subunit 2) is associated with immunodeficiency, including the CD4+ or CD8+ T cell dysfunction but normal function and number of NK cells [23]. CREBBP (CREB binding protein) is ubiquitously expressed in the transcriptional activation of transcription factors like Ap-2 [24]. CREBBP is a crucial regulator for maintaining cellular energy homeostasis by growth control [25]. GTF2H3 (general transcription factor IIH subunit 3) is an encoded protein, which is a subunit of the core TFIIH basal transcription factor and located in the nucleus [26]. GTF2H3 dysfunction is involved in the disorder of transcription-coupled nucleotide excision repair of damaged DNA, resulting in the genetic mutation of proteins and affecting DNA repair mechanism [27]. Such a pathological process induced by abnormal GTF2H3 expression may aggravate the immunodeficiency for individuals with Yang deficiency. Therefore, observation of Yang deficiency includes immune dysfunction tendency and energy deregulation. KAT2B, NFKB2, CREBBP, and GTF2H3 are represented as candidate markers for Yang deficiency.

On the other hand, JUNB, JUND, RAF1, NGLY1, PPP1R15A, and TNF in ME-green are the representative targets for the Yin deficiency group. More specifically, JUNB and JUND are the transcriptional factors and members of the JUN family. Both are functional components of the AP1 transcription factor complex [28, 29]. The common related pathological pathways for both JUNB and JUND are oxidative stress and T cell lymphoma [30–32]. RAF1 (RAF-1) is a cellular homolog of the viral RAF gene [33]. The phosphorylation of MEK1 and MEK2 could be stimulated by RAF1, which in turn activates the ERK1 and ERK2 to control cell cycle, apoptosis, and cell migration [34, 35]. Also, the related pathway of RAF1 is the oxidative stress pathway [36]. NGLY1 is an enzyme involved in the proteasome-mediated degradation of misfolded glycoproteins [37]. NGLY1 (N-glycanase 1) deficiency is related to neuropathy and the disorder of metabolism of protein, especially the dysfunction of endoplasmic reticulum [38, 39]. PPP1R15A (protein phosphatase 1 regulatory subunit 15A) is a member of GADD and MyD mammalian genes whose responsibility synergistically suppresses cell growth and endoplasmic reticulum-related cell death [40]. PPP1R15A is usually a target of ATF4 in endoplasmic reticulum stress [41]. TNF (tumor necrosis factor) encodes proinflammatory cytokines for the TNF family, which is primarily secreted by macrophages [42, 43]. TNF is capable of regulating lipid metabolism, cell growth, protein binding, and cytokine activity [44]. As TNF is a multifunctional cytokine with a wide spectrum of biological processes, suggesting that Yin deficiency may be resulted from the disturbance of essential substances of our body, such as blood and fluid, for supporting individuals with a healthy body constitution. Thus, JUNB, JUND, RAF1, NGLY1, PPP1R15A, and TNF might be the responsible targets for Yin deficiency. Features of Yin deficiency may include oxidative stress, dysfunction of the immune system, and abnormal lipid/protein metabolism.

**Figure 7:**
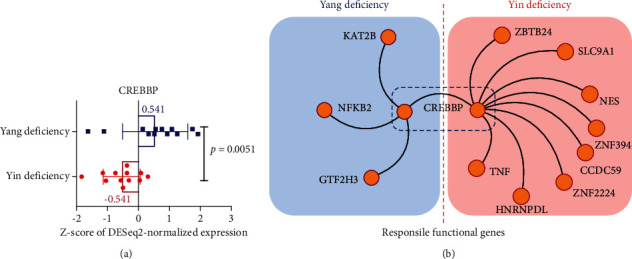
CREBBP is a shared key contributive gene regulating either the Yang or Yin deficiency group. (a) Z-score for DESeq2-normalized expression of CREBBP in either Yang or Yin deficiency. (b) Coexpressed and functional genes related to CERBBP in either Yang or Yin deficiency.

Notably, since the capacity of gene number of genome-wide genes is very large, it is worthy of discovering a shared and key functional target related to Yang or Yin deficiency by unsupervised analysis. Using WGCNA and functional analysis, CREBBP is coincidentally identified as a cofactor gene in regulating either Yin or Yang deficiency (Figure 6). For the biological characteristic of CREBBP, it is a key regulator for maintaining cellular energy homeostasis, which might be correlated with energy metabolism disorder in Yang or Yin deficiency symptoms. For the transcriptional analysis, the DESeq2-normalized expression of CREBBP in either the Yang or Yin deficiency group was significantly different (*p*=0.051) (Figure 7(a)). The mean value of CREBBP-related z-score is 0.541 (Yang deficiency) and −0.541 (Yin deficiency). These results suggested that the difference between a normalized expression of CREBBP in either the Yang or Yin deficiency group is around 1.082 standard deviation (SD) (|0.541| ∗ 2 = 1.082 SD), indicating the transcriptional expression ratio (1.082 SD) of CREBBP within the Yang or Yin deficiency group. In accordance with the gene functional analysis (Figure 6), CERBBP-associated coexpressed genes are illustrated in Figure 7(b). Taken together, our study might offer representative gene targets for supporting clinical traits of TCM theory-related Yang or Yin deficiency.

## 5. Conclusion

On the basis of Chinese medicine theory and R-dependent machine learning, our study provided the transcriptomic profile of subjects with Yang or Yin deficiency body constitution by reanalyzing RNA-Seq data in GEO. The main merits of this study are as follows: (1) by reanalyzing RNA-Seq data, we identified the responsible targets for Yang deficiency (KAT2B, NFKB2, CREBBP, GTF2H3) and Yin deficiency (JUNB, JUND, NGLY1, TNF, RAF1, PPP1R15A) in Chinese medicine theory; (2) functional annotations for the representative targets in both body constitution (Yang or Yin deficiency) were provided; and (3) R-based machine learning approaches used in this investigation might be further adopted for unveiling the potential genetic polymorphisms related to the Chinese medicine theory. However, the limitation of this study is that, although we identified the key gene targets for the body constitution with Yang or Yin deficiency, target validation based on clinical and basic experimental investigations should be further provided for strengthening the findings in this study.

## Figures and Tables

**Table 1:** Clinical symptom of Yang or Yin deficiency.

	Yang deficiency	Yin deficiency
Main features	Cold intolerance	Heat intolerance
	Fluid retention, chills, fat, nocturia	Dryness, thirst, insomnia, emaciation
Tongue	Prefer pale tongue	Prefer red tongue
Pulse	Prefer slow pulse	Prefer rapid pulse

## Data Availability

The data used to support the findings of this study are included within the article, supplementary information files, and NCBI GEO database (GSE87474).

## Supplementary Materials

Supplementary Materials Supplementary Data 1: normalized data set of GSE87474 by DESeq-2 in R. Supplementary Data 2: the value of module eigengenes of filtered genes in the Yang deficiency group. Supplementary Data 3: the value of module eigengenes of filtered genes in the Yin deficiency group.
